# Densification Kinetics and Structural Evolution During Microwave and Pressureless Sintering of 15 nm Titanium Nitride Powder

**DOI:** 10.1186/s11671-016-1316-x

**Published:** 2016-02-24

**Authors:** Ostap Zgalat-Lozynskyy, Andrey Ragulya

**Affiliations:** Frantsevich Institute for Problems of Materials Science, 3, Krzhizhanovsky str., 03680 Kiev, Ukraine

**Keywords:** Microwave sintering, Nanoparticles, Activation energy, Grain growth, Grain boundary, TiN

## Abstract

Microwave sintering (MWS) of commercially available 15-nm-size nanocrystalline TiN powder was studied. Densification kinetics and grain growth mechanisms of nano-TiN were evaluated using non-isothermal heating up to 1500 °C with variable heating rates. A true nanocrystalline ceramic with ~80-nm-size grains and 94.5 % theoretical density was obtained via MWS consolidation at 1400 °C. At higher temperatures, however, an uncontrolled grain growth and a formation of bimodal microstructure were noticed. A temperature dependence of grain growth suggested grain boundary sliding as a primary mechanism of densification below 1100–1200 °C. An activation energy of nano-TiN densification under MWS varied from 26 ± 3 kJ/mol at the initial stage of sintering (900–1200 °C) to 162 ± 22 kJ/mol at higher temperatures. In addition, a relationship coupling microstructural characteristics (grain size, grain boundary) with mechanical properties of titanium nitride ceramics obtained via both microwave and pressureless sintering techniques was discussed.

## Background

Since 2000, when H. Gleiter first formulated a paradigm for manufacturing nanostructured materials from nanoparticles [1], the research activities in this area have been rapidly developing. The mechanical and electrical properties of nanostructured materials, however, were found to be more sensitive to their microstructural parameters (grain and pore size distribution), compared to conventional coarse-grained counterparts. Theoretical predictions and experimental findings confirmed the appearance of enhanced physical properties in nanostructured materials when grains become less than 100 nm in size [1–3]. Some nanostructured materials demonstrated so-called size effect when fine grains enhanced material properties, such as toughness and hardness [3–6]. A discovery of “size effect” stimulated the development of several new powder consolidation techniques, specifically designed for manufacturing dense nanostructured materials [3–7]. Among them, field-assisted sintering techniques such as spark plasma sintering (SPS) and microwave sintering (MWS) were intensively developed [4, 5, 7–9]. The microwave sintering is a rapid rate sintering technique suitable for various classes of materials including nanomaterials. MWS utilizes an ability of materials to directly absorb microwave energy resulting in self-heating. The effect of particle size and porosity on heating of conductive materials (metallic particulate compact) in a 2.45-GHz multimode microwave furnace was investigated by Agrawal et al. [8, 10, 11]. The experiments with copper powders in microwave furnaces suggested that samples with higher porosity and smaller particle sizes were able to heat more rapidly [10–12] compared to the conventional heating conditions. In the microwave process, the microwave energy is absorbed directly by a sample, leading to a uniform volume heating without thermal gradients within the material. In addition, the microwave energy is capable of simultaneously heating large and small samples uniformly with high heating rates. This becomes especially important for nanostructured materials where undesired grain growth can be minimized by rapid heating with short thermal cycles [2, 6, 7]. Thus, a consolidation of nanocrystalline powders via microwave sintering has a great potential to obtain materials with grains sizes below 100 nm.

During microwave-enhanced treatment of nanocrystalline powders (high-specific surface area of which is the main absorber of microwave energy), the surface-related defects may influence mass transport mechanisms such as a grain boundary diffusion [13–15]. In addition, due to the increased surface area, the nanocrystalline powders generally interact more effectively with microwaves and can demonstrate the reduction of a material’s melting temperature [9, 11, 13]. This is an additional incentive to continue the investigation of the microwave processing of nanomaterials as well as the reason to apply the MWS method for production of nanocomposites with enhanced properties.

In this paper, a possibility to use microwave sintering for manufacturing dense nanostructured titanium nitride ceramics was studied. A conventional sintering technique was used here for a comparison purpose. A focus of the paper was made on revealing characteristic features of densification kinetic, ceramic microstructure, and mechanical properties of nano-TiN ceramic consolidated under different techniques.

## Methods

**Fig. 1:**
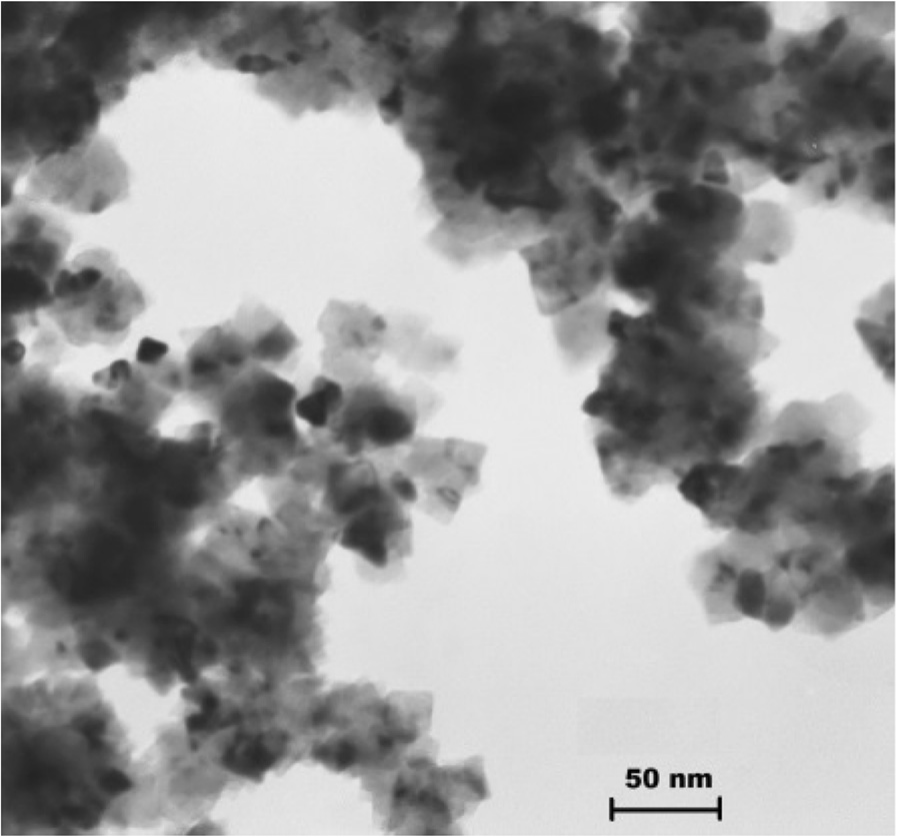
TEM microphotograph of TiN nanopowder

A commercially available titanium nitride nanocrystalline powder (HC Stark GmbH, Germany) with an average particle size ~15 nm was used to evaluate the formation of ceramic microstructure during both microwave and pressureless sintering. A transmission electron microscope (TEM) image of TiN nanopowder is shown in Fig. 1.

A microwave multimode furnace (1 kW, 2.45 GHz) capable of heating various materials up to 1500 °C both in vacuum (10^−3^ Torr) and in gas (nitrogen, argon) atmospheres was used in this study [9]. Due to the fact that titanium nitride can directly absorb microwave energy at 2.45 GHz, no additional heating elements or microwave susceptors were used. Sintering temperature was monitored both by a shielded S-type thermocouple and by a digital pyrometer AR 922. Based on the recorded temperature-time data, the heating rate of TiN samples during microwave sintering was estimated to be in the range from 30 to 45 °C/min.

Pressureless sintering (PS) of TiN nanopowder was carried out using high-temperature gas-vacuum furnace “Centorr VI Series 15” (Centorr Vacuum Industries, USA). The heating rate of 45 °C/min was used to obtain ceramic samples (PS45) with structural characteristics similar to that of the samples sintered in microwaves.

Both PS and MWS experiments were performed according to the following schedule: titanium nitride green pellets 15 × 15 × 4 mm in size (three pellets in a batch) were heated in nitrogen to temperatures from 900 °C to 1500 °C with 100 °C steps followed by a rapid cooling. The samples sintered at different temperatures were used for microstructural analysis and mechanical tests. Microstructure and grain boundary analysis of sintered TiN ceramic samples at 1400 °C and 1500 °C was performed by a Scanning Transmission Electron Microscopy (STEM) HD 2700 (Hitachi, Japan).

Oxygen content was certified using chemical analysis (determination of oxygen content by reducing extraction as per GOST 27417–87). For initial 15-nm TiN nanopowder, oxygen content was 0.8 wt.%. After consolidation fixed a slight increase of oxygen content to 1.4 wt.% (PS) and 1.2 wt.% (MWS), formation of additional oxide or oxynitride phases was not determined on XRD.

A final density of the consolidated materials was measured by an Archimedes method in de-ionized water at room temperature. A qualitative X-ray diffraction analysis (XRD) was performed using both XRD-7 (Seifert-FPM, Freiberg, Germany) and DRON-3M (Russia) diffractometers under Cu_Kα_ radiation. A size of crystallites in sintered ceramic samples was estimated using Scherrer equation and calculated average for three pellets in the batch. The average grain size was measured from TEM micrographs using a line-intercept method (55 grains). Vickers hardness and fracture toughness were measured at 50 g and 10 kg load conditions by both PMT-3 (LOMO, Russia) and MMT-3 (Buehler, USA) hardness testers, respectively. Nanohardness was measured by a “Micron-Gamma” (NAU, Ukraine) nanohardness tester equipped with Berkovich indenter under 20 cN load conditions.

## Results and Discussion

### Sintering Kinetics of TiN Nanopowder

**Fig. 2:**
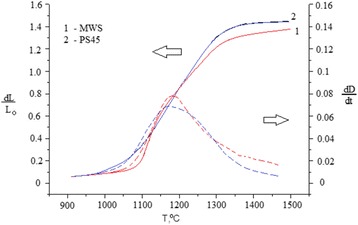
Temperature dependence of linear densification and linear densification rate for nano-TiN sintered by MWS and PS

The linear shrinkage kinetic curves for nano-TiN were recorded during MWS and PS processes and are shown in Fig. 2. The samples obtained via pressureless sintering with 45 °C/min heating rate (PS45) reached a full density (~99 % of theoretical value of 5.43 g/cm^3^) at about 1400 °C. The shrinkage rate exhibited a maximum value of 0.075 1/s at 1230 °C. The densification of nano-TiN via MWS condition proceeded much faster compared to PS. The maximal shrinkage rate 0.086 1/s was achieved only at 1242 °C most likely due to unstable heating with variable heating rates from 30 to 45 °C/min. Instability of the heating rate during MWS process was also observed previously for TiN-based nanocomposites. Variability of the heating rate can be explained by peculiarities of microwave absorption by the material due to the evolution of material’s density and pore microstructure [9, 11–13]. The maximal relative density about 96 % of theoretical was achieved only at ~1500 °C. Titanium nitride is an electrically conductive material, so when the relative density of the ceramic samples reaches ~80 %, the reflection of microwave energy increases rapidly, resulting in substantial deceleration of the densification process. Preserving of open porosity (channel porosity) up to the high temperatures should significantly retard the grain growth. Moreover, for microwave processing of conductive materials, remaining porosity stimulates uniform heating of the sample. The effect of porosity and particle size on densification of conductive powders (copper) was investigated by Mondal et al. [11]. The authors [11] note that powder compacts with higher porosity and smaller particle sizes interact more effectively with microwaves and are heated more rapidly. In general, to achieve high densities during MWS of conductive materials, some special additives to the material or a hybrid MW heating arrangement may be required [8, 9].

**Fig. 3:**
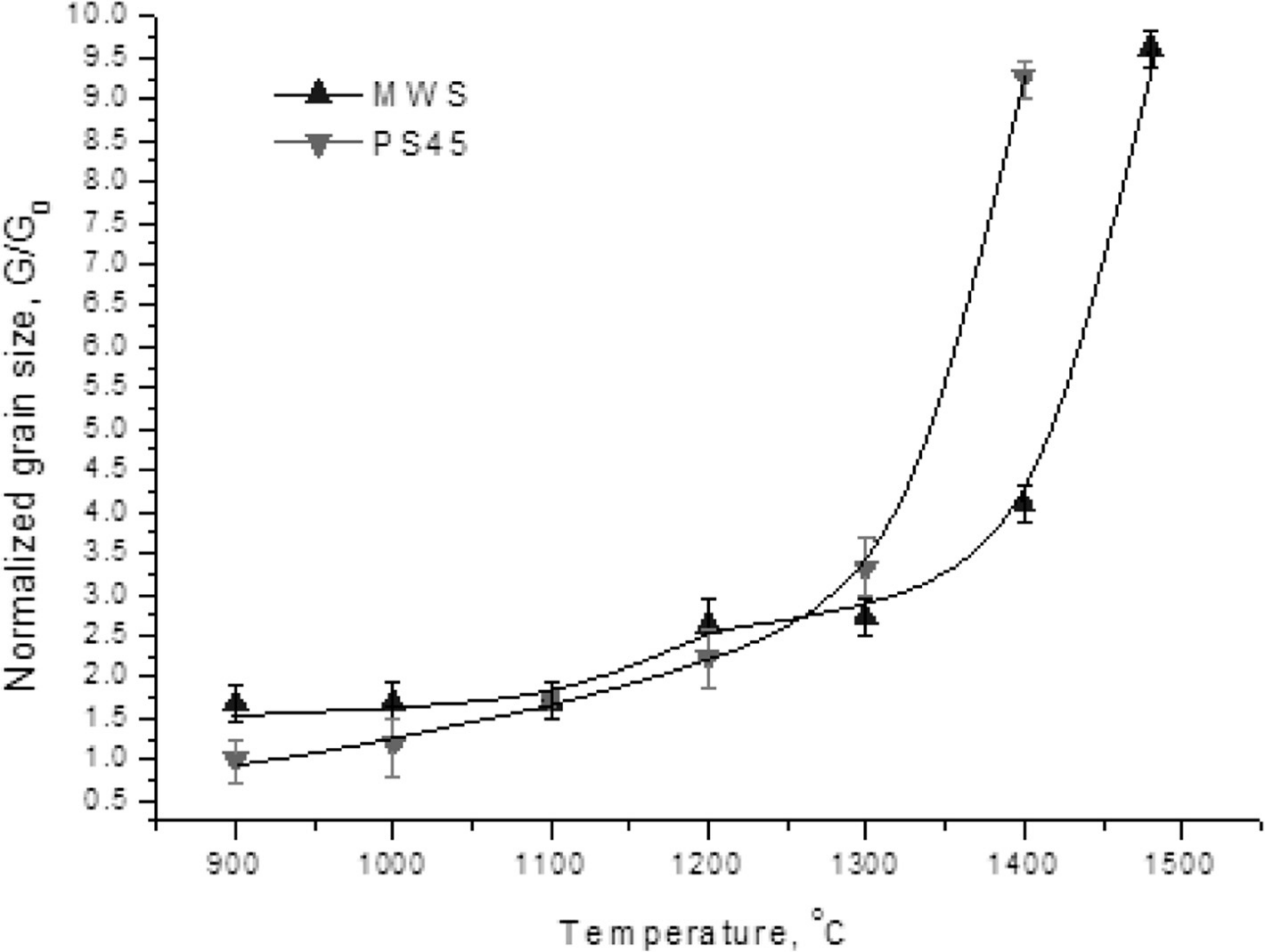
The dependence of normalized grain size vs. sintering temperature

**Fig. 4:**
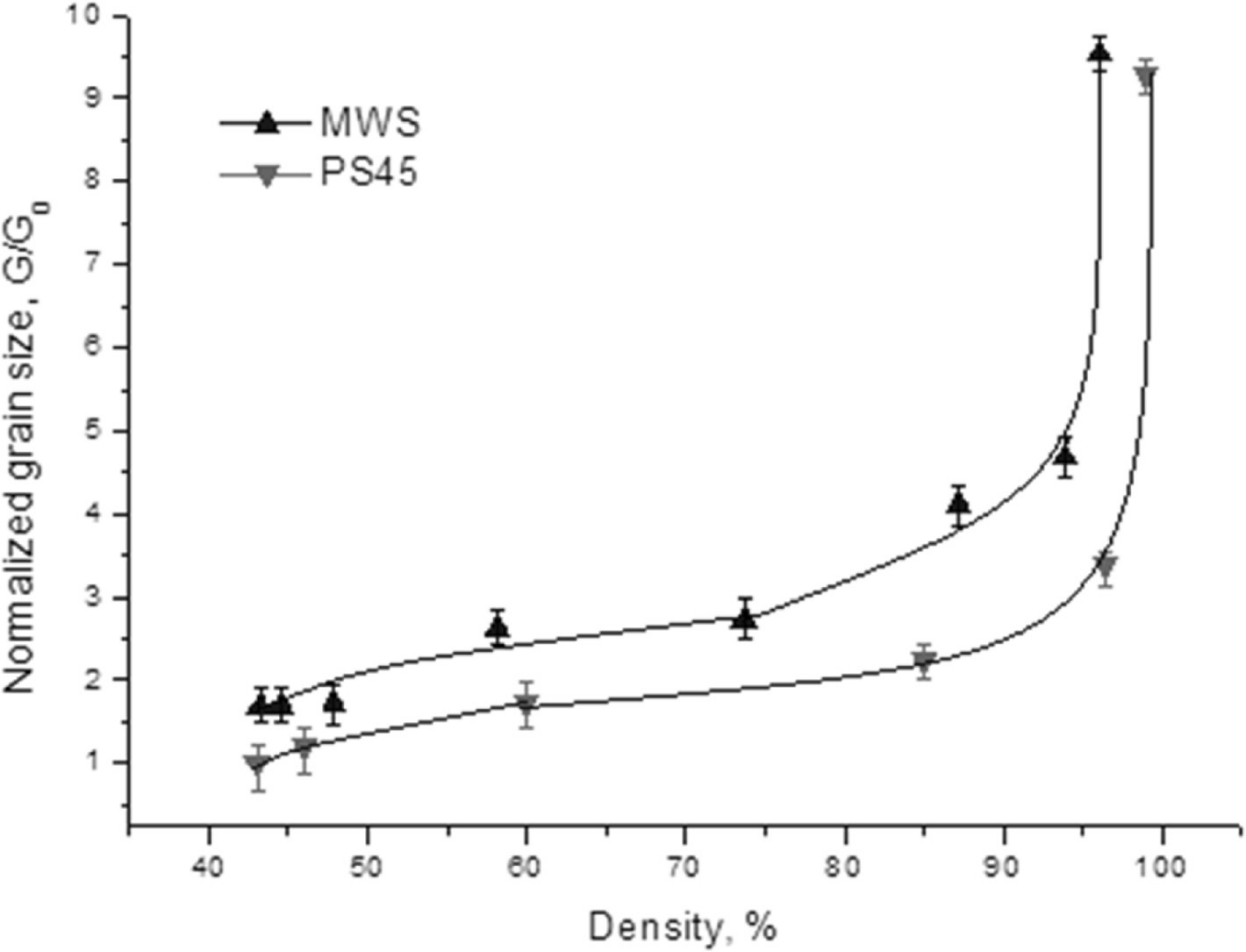
A dependence of normalized grain size vs. ceramic density

Figure 3 shows an estimation of crystallite sizes for nano-TiN ceramic samples heated up to different temperatures by two consolidation techniques. Two distinct stages of densification were observed for both consolidation techniques: (i) below 1200 °C—high densification rate without a significant grain growth and (ii) above 1300 °C—rapid grain growth accompanied by a slow densification rate (Figs. 2 and 3). The analysis of temperature dependence of normalized grain size (Fig. 3) and linear densification rate (Fig. 2) suggests that during the initial stage of sintering (*T* < 1100–1200 °C), the primary mechanism of densification could be a grain boundary sliding. As it was discovered by Ashby [16], the densification rate under the grain boundary sliding mechanism is about 10 times higher than under a diffusion-viscous flow mechanism. At the same time, the grain growth under the grain boundary sliding mechanism remains the slowest among other possible mechanisms (*G*/*G*_0_ < 2) (Fig. 4).

Moreover, at temperatures below 1100 °C, nano-TiN consolidated via MWS exhibited stable grains with sizes ~28 ± 4 nm contrary to PS45 (Fig. 3). This could be an evidence of a domination of grains rotation and accommodation densification mechanism during the low-temperature stage of MWS consolidation.

The analysis of TiN grain size evolution under the non-isothermal microwave heating conditions revealed a suppression of the grain growth (*G*/*G*_0_~2.5) in the temperature range from 1200 to 1350 °C (Fig. 3). This deceleration of the grain growth coincides well with the temperature range of fast densification during heating with a constant heating rate (Fig. 2 curve 1).

During PS, the intensive densification proceeded up to ~95 % of theoretical density without the considerable grain growth (*G*/*G*_0_ < 2.5) (Fig. 4). The results suggest that the intensive grain growth (*G*/*G*_0_ ≥ 9) starts right after the completion of the active densification stage, i.e., in the 1200–1300 °C temperature range. Above 1300 °C, the porosity became closed (6–8 %) and the uncontrolled grain growth took place.

Similar to pressureless sintering, the grain growth during MWS process of nano-TiN can be characterized by almost a linear trajectory up to ~95 % of theoretical density (Fig. 4). However, at densities higher than 85 %, the microstructure of the material became inhomogeneous due to the influence of the temperature gradient within the material caused by a partial reflection of microwave energy by a conductive sample’s surface. In the temperature range from 1300 to 1400 °C, the porosity reduces from 13 to 5 % (predominantly open porosity).

It is worth noting that a difference between the normalized grain size in both processes is substantial (Fig. 4). The low-temperature coalescence occurred more intensively under MWS rather than PS conditions. Most likely, at the initial stage of microwave sintering, the actual temperature in the contact zones (necks) was much higher than the average temperature within the volume of the material. The particle surface could melt and intensify a surface diffusion at contacts between particles. This could lead to particles coarsening via a coalescence mechanism (Fig. 4).

Based on these results, it is possible to conclude that the best ratio between the densification rate and the grain growth (highest densification rate at lowest grain growth) for PS was achieved in the temperature range from 1100 to 1250 °C. For MWS, on the other hand, the inhibition of grain growth was found at temperatures up to 1350–1400 °C.

Additionally, an analysis of the grain growth kinetic was performed using an Arrhenius-type equation:

1 $$ d\left({G}^{\mathrm{n}}-{G_0}^{\mathrm{n}}\right)/\mathrm{d}\mathrm{t}=k $$

where *G*, *G*_0_, and *n* (*n* ≥ 2) are an average grain size at time *t*, an average initial grain size, and a grain growth exponent, respectively [17, 18]. *k* is a constant that satisfies the following equation:

2 $$ k={k}_0\; \exp \left(-Q/\mathrm{R}\mathrm{T}\right), $$

where *Q* is an activation energy, *T* is a temperature in Kelvin, *k*_0_ is a pre-exponential rate constant, and *R* is a gas constant. For non-isothermal, *T* = *T*_0_ + *ht* where *T*_0_ is the initial temperature, *T* is the current temperature, and *h* is the heating rate.

3 $$ d\left({G}^n-{G}_0^n\right)/\mathrm{d}\mathrm{t}={k}_0 \exp \left(-\frac{Q}{\mathrm{Rht}}\right), $$

Conventionally, the activation energy *Q* could be obtained from the Arrenius equation by plotting a ln *d*(*G*^*n*^ − *G*_0_^*n*^)/dt vs. 1/*T*.

The rate of grain growth, *dG*/*dt*, can be derived as [19, 20]:

4 $$ \left(\frac{1}{G}\frac{dG}{dt}\right)=\frac{K_2}{G^n{\left(1-\rho \right)}^l} $$

where *ρ* the sample density, *l* the sample size. Then, in logarithms:

5 $$ Ln\left(\frac{1}{G}\frac{dG}{dt}\right)=Ln\;{K}_2- nLnG-lLn\left(1-\rho \right) $$

**Fig. 5:**
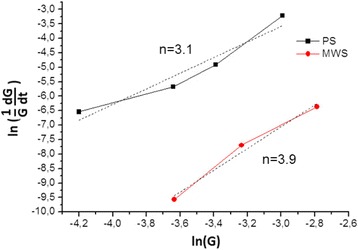
Plots of ln((1/*G*) · (*dG*/*dt*)) vs. ln(*G*) for nano-TiN consolidated at the temperatures 1000–1300 °C

From the slope of the line of ln ((1/*G*) · (*dG*/*dt*)) vs. lnG, the grain growth kinetic exponent (*n*) was determined [20]. The grain growth kinetic exponents (*n*), estimated by this method at the temperature range 1000 to 1300 °C were 3.9 and 3.1 for MWS and PS, respectively (Fig. 5). The growth exponent *n* obtained for the PS and MWS indicates high resistance against grain growth for nano-TiN at the temperatures up to 1300 °C.

The exponent 3.1 could be valid for combination of two mechanisms (2 for pure boundary mobility and 4 in the case of pore’s pinning the grain boundaries) with domination of pinning while the porosity exists. The *n* = 3.9 value assumed domination of grain growth mechanism attributed to grain boundary retardation by inclusions or pores. At higher temperatures, normal grain growth dominates due to a grain boundary diffusion (*n* = 2).

Authors [6] reported comparable data about grain growth of nano-TiN during PS (55 °C/min). Estimated grain growth exponent and activation energy for nanocrystalline powder 40 nm were 3.2 and 400 kJ/mol, respectively. Considering the fact that up to 1300 °C, the TiN samples held nanometer-sized grains and material characterized by a large volume of grain boundary fraction, the grain growth through the boundaries diffusion is much expected than that via conventional lattice diffusion.

**Fig. 6:**
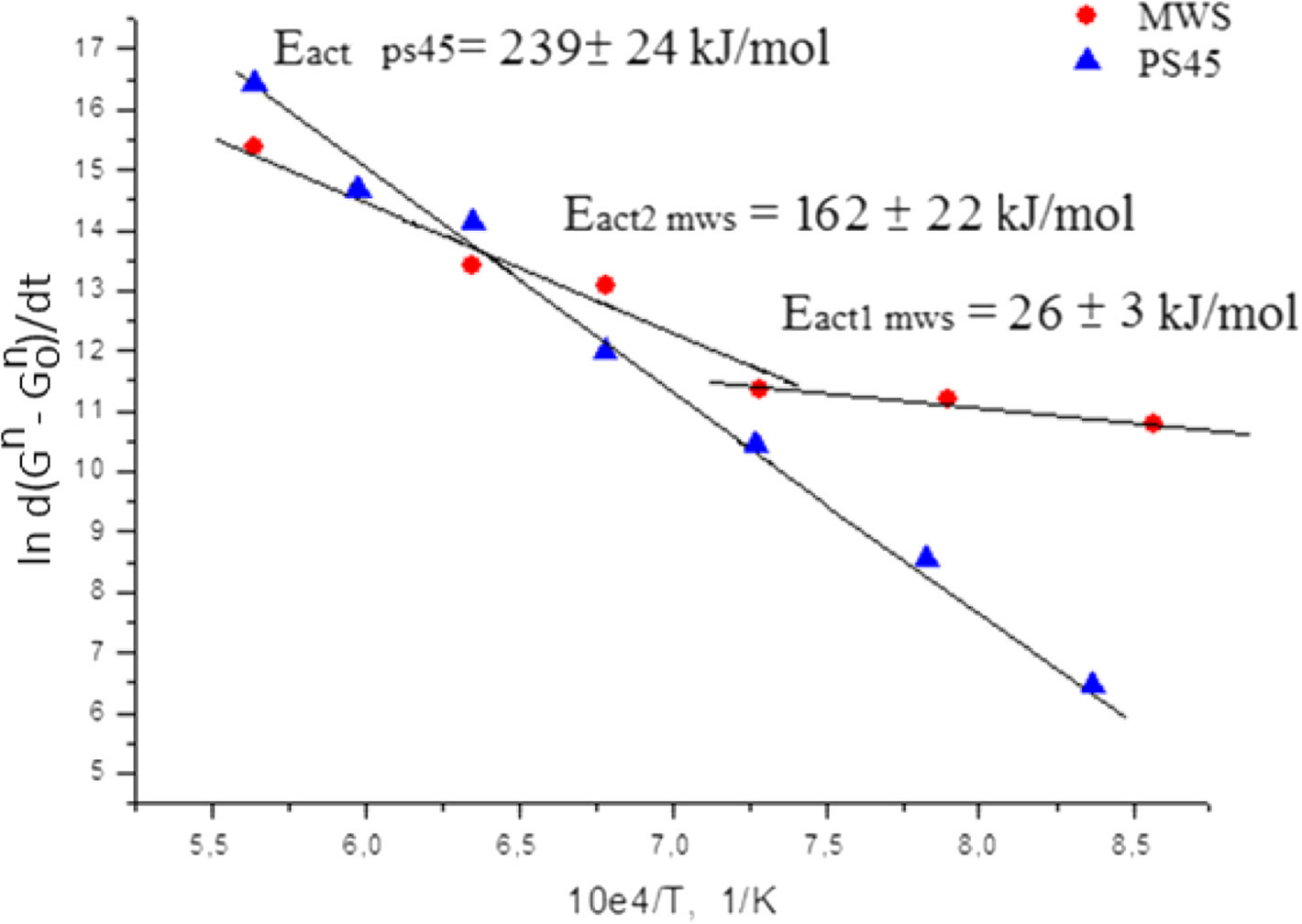
Arrhenius plots for the grain growth of nanocrystalline TiN

Figure 6 shows a reciprocal temperature dependence of the effective activation energy estimated for both consolidation techniques. The effective activation energy for PS45 remained constant in the entire temperature range and was estimated to be ~239 ± 24 kJ/mol. A constant level of the activation energy suggests only one prevailing densification mechanism. At the same time, MWS exhibited two clearly distinctive stages of densification with two different values of the activation energy. At lower temperatures, around 900–1100 °C, the estimated activation energy was found to be ~26 ± 3 kJ/mol. This small level of the activation energy suggests the formation of a liquid phase at particle contact points which facilitates sliding of particles without a significant grain growth. Authors [13] observed similar effect of material melting during MWS of nanocrystalline titanium nitride. Moreover, nano-TiN demonstrated a tendency to the formation of strong agglomerates at the temperatures as low as 350 °C [9]. At the same case at PS, this process starts at higher (650–800 °C) temperatures. This can be attributed to high sinterability of investigated nanopowders in microwaves. At higher temperatures around 1200–1500 °C, the activation energy increased up to 162 ± 22 kJ/mol level, which could be an indication of a slight contribution from the volume diffusion [6, 21, 22]. So, the two distinct levels of the effective activation energy suggest an existence of a rapid transition from the region dominated by the grain boundary diffusion with particle rearrangement to the region dominated by the volume diffusion.

#### Microstructural Investigation of Nano-TiN Consolidated by MWS and PS

As discussed above, both MWS and PS45 consolidation techniques led to the formation of ~95–97 % of theoretical density in nano-TiN samples at 1400 °C (Fig. 2). Subsequent heating of the samples to 1500 °C resulted only in a minor densification with simultaneous uncontrolled grain growth (Fig. 6). A transmission electron microscopy was used to characterize microstructure of the samples consolidated both at 1400 and 1500 °C to prove theoretical considerations about grain growth at pressureless and microwave sintering. Moreover, the obtained information about grain sizes and grain boundaries of nano-TiN samples was used for the prediction of mechanical properties of the material consolidated at different temperatures.

**Fig. 7:**
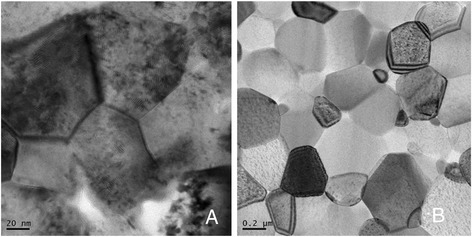
BF-STEM micrographs of nano-TiN ceramics consolidated by MWS at the temperatures **a** 1400 °C and **b** 1500 °C

Figure 7 shows bright-field scanning transmission electron microscopy (BF-STEM) microphotograph images of selected samples consolidated at 1400 and 1500 °C by MWS technique. The smallest grain size (*d* ~ 80 nm) was observed in the MWS sample consolidated at 1400 °C (Fig. 7a). The sample demonstrates uniform structure with grain size average deviation ±6 %. At 1500 °C, the MWS processed sample exhibited the abnormal grain growth (Fig. 7b). The microstructure of MWS sample at this temperature exhibited non-uniform grain size distribution from 150 to 400 nm. It could be a result of inhomogeneous heat distribution within the volume of the sample due to the expression of skin affect for bulk TiN while the remaining porosity becomes predominantly closed.

**Fig. 8:**
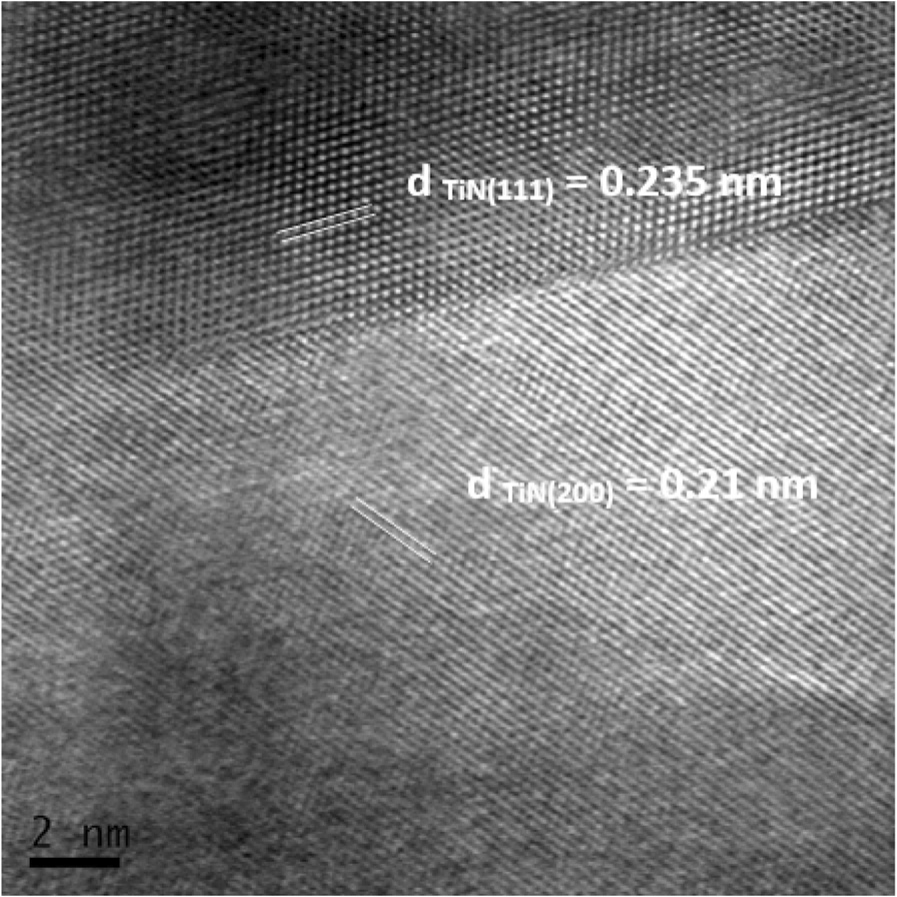
High-resolution image of grain boundary for MWS sample consolidated at 1400 °C

The 80-nm grain size TiN samples consolidated by MWS at 1400 °C exhibited well-connected grain boundaries. This was confirmed by a high-resolution observation (Fig. 8). Grain boundaries were found to be planar, low contaminated, and well developed without any amorphous layers.

**Fig. 9:**
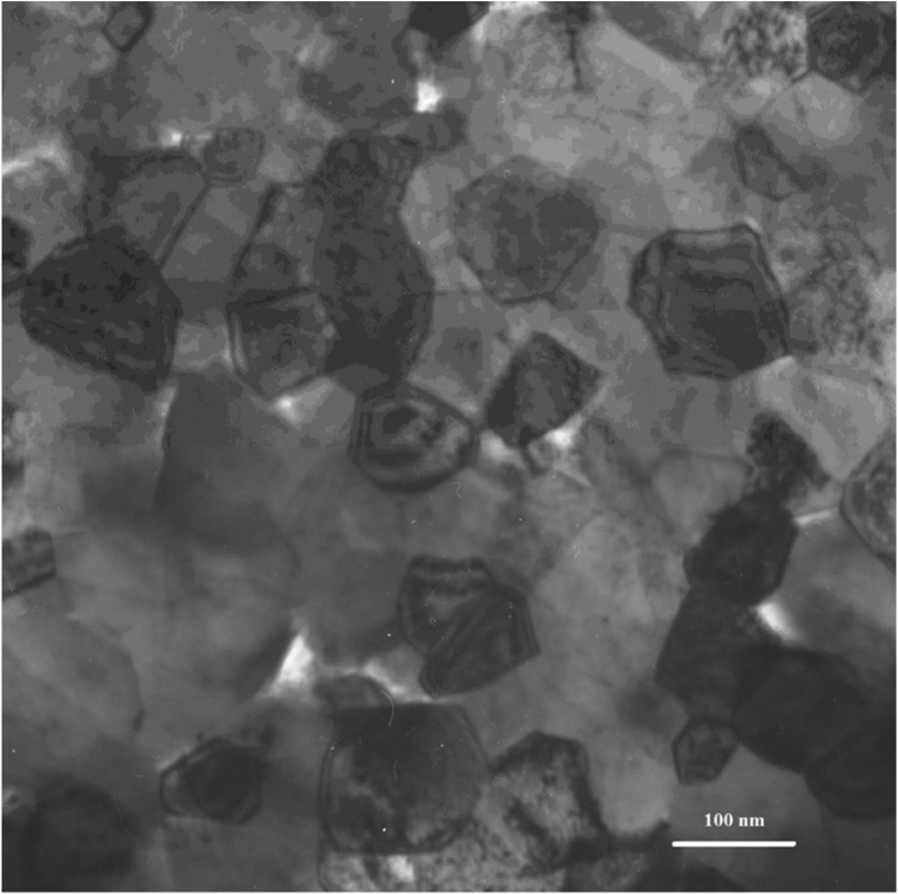
TEM micrograph of nano-TiN ceramics consolidated by PS45 at the temperature 1400 °C

PS45 sample sintered at 1400 °C exhibited a homogeneous microstructure with the average grain size ~155 nm. This is about two times higher as compared to the average grain size of MWS sample consolidated at the same temperature (Fig. 9).

#### Mechanical Properties of Nano-TiN Consolidated by MWS and PS

To estimate a relationship between grain size of consolidated ceramic and its mechanical properties, a modified Hall-Petch Eq. (6) was used [3]. The calculated data was compared with the data obtained during hardness (Vickers hardness and nanohardness) tests of nano-TiN materials:

6 $$ H={H}_{\mathrm{g}}.\kern1em \left( 1-f\right)+{H}_{\mathrm{g}\mathrm{b}.\kern1em }f $$

where *H*_g_ and *H*_gb_ are a hardness of grains and grain boundaries, respectively, and *f* is a volume of grain boundaries.

An integrated formula (7) was proposed by authors [3]. The formula takes into account not only grain sizes of the material but also the volume of grain boundaries, which is the critical factor for nanomaterials, the hardness characteristics of coarse grain counterparts and an extra parameter describing the arrangement of triple junctions. The effect of triple junctions on the local stress distribution could be considered by using disclination theory.

7 $$ H={H}_{0g}+{k}_g\cdot \left[\frac{{\left(d-\delta \right)}^3}{d^3}+\frac{d^3-{\left(d-\delta \right)}^3}{d^3}\cdot \left(\frac{ \ln \frac{\vartheta \cdot d}{r_0}}{ \ln \frac{\vartheta \cdot {d}_c}{r_0}}\right)\right]\cdot {d}^{-\raisebox{1ex}{$1$}\!\left/ \!\raisebox{-1ex}{$2$}\right.} $$

where *d* is the average diameter of grains, *ϑ* is the number factor less than 1, *δ* is the grain boundary width, and *r*_0_ is the radius of dislocation core [3].

The critical transition grain size (*d*_c_) can be derived as

8 $$ {d}_{\mathrm{c}}=\left(\left(G\cdotp b\right)/\left(\left(1-\nu \right)\cdotp H\right)\right), $$

where *G* is the shear modulus, *b* is the Burgers vector, *ν* is the Poisson’s ratio, and *H* is the measured material hardness.

The dependence of hardness vs. grain size had been previously calculated for titanium nitride and revealed that the maximal hardness (around 32 GPa) can be achieved for the material with grain size ~10 nm. Ten-nanometer grain size corresponds well to the critical grain size with maximum pile-up dislocations in titanium nitride [7]. The properties of MWS and PS45 consolidated materials are summarized in Table 1.

**Table 1:** Mechanical properties of titanium nitride

Consolidation method	T, °C	Density, %	Grain size, nm	Calculated hardness, GPa	HV, GPa	Nanohardness, GPa	E, GPa
MWS	1400	94.5	80	25	20.8 ± 2.4	23.09 ± 1.56	283 ± 21
MWS	1500	96	200	22	21 ± 2.2	24.09 ± 1.6	301 ± 18
PS45	1400	97.5	155	23	21.9 ± 1.9	25.89 ± 3.04	314 ± 45

As calculated from Eq. 7, the maximum hardness of 25 GPa can be achieved for the MWS sample with the average grains size ~80 nm sintered at 1400 °C. The calculated value of hardness is in a good agreement with measured nanohardness ~23.09 ± 1.56 GPa but differs from the measured Vickers hardness ~20.8 GPa. MWS samples sintered at 1500 °C and PS45 samples sintered at 1400 °C exhibited Vickers hardness ~21–22 GPa, which is in a good agreement with the calculated values. However, measured values of nanohardness are still higher compared to the calculated ones.

Some differences in hardness results may be explained by the effect of porosity in nano-TiN as well as differences in measurement techniques [6, 7, 23]. Since the estimated volume of the material involved in the nanohardness test is only about 8–10 grains, the influence of porosity is negligibly low. The Vickers hardness test requires much larger volume of the material, so the residual porosity may affect the measured hardness and elastic modulus of the material.

On the other hand, authors [23] investigated the influence of Berkovich and Vickers indenters’ geometry on indentation test results of bulk materials. It was found that the measured hardness not only is material sensitive (phase content, structure, porosity, etc.) but also depends on the indenter geometry. In the case of bulk materials, a maximum value of equivalent plastic strain and hardness is higher for the Berkovich indenter than for the Vickers.

## Conclusions

The analysis of the densification kinetic and the grain size evolution of nano-TiN under non-isothermal microwave and pressureless heating conditions revealed some differences during the initial stages of material processing. The high densification rate without significant grain growth for the pressureless sintering of nano-TiN was achieved in the temperature range from 1100 to 1250 °C. At the same time, for MWS, inhibition of grain growth was prolonged up to the 1350–1400 °C.

The analysis of the evolution of normalized grain size with temperature suggested that at the initial stages of microwave sintering below 1100–1200 °C, the main mechanism of densification could be the grain boundary sliding. For temperature range below 900–1100 °C, the activation energy for microwave-sintered nano-TiN was estimated at 26 ± 3 kJ/mol level. Such a low level of the activation energy could be explained by the formation of a liquid phase at contacts between particles facilitating a densification via particles sliding without a significant grain growth. At the higher temperatures, however, the uncontrolled grain growth took place, and, in the case of MWS, the formation of bimodal structure at 1500 °C was observed.

The smallest grain size (*d* ~ 80 nm) was recorded in MWS samples consolidated at 1400 °C to the density 94.5 %. At the same time, the pressureless sintered sample at 1400 °C demonstrated higher density, ~97.5 %, with larger average grains size ~155 nm.

A theoretical hardness (25 GPa) calculated for the MWS samples with average grains size ~80 nm was in a good agreement with the measured nanohardness 23.09 ± 1.56 GPa but was different from Vickers hardness, ~20.8 GPa. MWS and PS45 samples consolidated at 1500 and 1400 °C, respectively, demonstrated Vickers hardness ~21–22 GPa, which is in a very good agreement with the calculated values. Some differences in measured and calculated values of the hardness data could be explained by the effect of residual porosity.

Finally, comparing the PS and MWS consolidation of 15 nm TiN, it was noted that the pressureless sintering was capable to produce a near fully dense fine-grained material at the temperature ~1300 °C. On the other hand, the main advantage of the microwave sintering technique compared to the pressureless sintering is the ability to prolongate the period with inhibited grain growth up to 1400 °C and produce nanocrystalline TiN ceramic with “clean” grain boundaries. This is important for designing TiN-based composite nanomaterials, where high consolidation temperatures are required.

## Abbreviations

BF-STEM: bright-field scanning transmission electron microscopy
MWS: microwave sintering
PS: pressureless sintering
